# Orofacial pain of cardiac origin, serial of clinical cases

**DOI:** 10.4317/medoral.17689

**Published:** 2012-02-09

**Authors:** José López-López, Maria J. Adserias-Garriga, Laia Garcia-Vicente, Enric Jané-Salas, Eduardo Chimenos-Küstner, Damián Pereferrer-Kleine

**Affiliations:** 1 MD, PhD, Medical Doctor Specialist in Stolmatology. Professor of Oral Medicine. Faculty of Dentistry. University of Barcelona; 2 PhD. Dentistry. Professor of Oral Medicine. Faculty of Dentistry. University of Barcelona; 3 DDS. Dentistry. Máster in Oral Medicine. Faculty of Dentistry. University of Barcelona; 4 MD, PhD, Medical Doctor. Specilist of Cardilogy. Hospital Germans Trias i Pujol. Badalona (Barcelona)

## Abstract

Objective: To determine the clinical characteristics of the orofacial pain of cardiac origin in patients visited when doing a treadmill exercise test, at the cardiology service of the Can Ruti Hospital in Badalona (Barcelona, Spain). Study design: The sample of that study included thirty patients visiteding when doing a treadmill exercise test, at the cardiology service. The questionnaire has been asked to a sample of 30 patients. Results: Eleven of the 30 patients included in this study presented craniofacial pain before or during the cardiac seizure. The location of the pain was bilateral, non-irradiated at the mandible in all cases. The intensity of the pain was from slight to severe. The frequency of the appearance of the pain was paroxysmal in 8 cases and constant in three cases, and the duration was from a few hours to a maximum of 14 days. Discussion: The cardiac pain in craniofacial structures is usually bilateral, compared to odontogenic pain which is always unilateral. The pain of cardiac origin is considered atypical because of its location, but about the 10 % of the cases, the cardiac ischemia has its primary manifestation in orofacial structures. Conclusions: Eleven patients referred a bilateral non-irradiated mandibular pain, with intensity from slight to severe, and with a paroxystic frequency in eight cases and a constant frequency in three cases. Just one patient referred pain during the treadmill exercise test. In all cases the pain disappeared after the cardiac surgery or the administration of vasodilators.

** Key words:**Orofacial pain, toothache, angina pectoris, mandibular pain, myocardial infarction, angina pectoris.

## Introduction

The cardiac ischemia constitutes one of the main causes of death in the adult population (1). The most characteristic clinical symptom of that pathology is sub sternal pain irradiating to shoulders, arms and neck. The characteristics that define the coronary pain are: location, irradiation, frequency, intensity, precipitating factors and the circumstances that relief it. Usually, the cardiac patients describe the anginous pain as an oppression located in most cases at the retrosternal region, and it can irradiate arms, neck and jaw. But, some cases are an exception, being the symptoms of the cardiac process just a difficulty to breath or a painful sensation located only in the neck, jaw, arms, or even in the wrist (2-7).

In some cases the pain can irradiate jaws and teeth (2,3). The cause of the cardiac pain referred to the orofacial region can be explained by the convergent mechanisms of the trigeminal complex. Being the Vagus nerve an important mediator of that pain (8,9). In addition, it has been reported that cardiac afferences and somatic efferences from the upper limb, the thorax and the face converge all in the spinothalamic neuronal tract of the central nervous system (4). It is known that the most common orofacial pain has a dental, periodontal or musculoskeletal cause. Nevertheless, the patient can refer pain in that region and the cause of the pain can be located distantly. This type of pain is called heterotopic pain of cardiac origin (2-5). The lack of an accurate diagnosis when these symptoms appear can lead to unnecessary dental treatment. There are some clinical cases published where the patients received unnecessary dental treatments such as dental extraction or the administration of analgesic for a wrong diagnosed temporomandibular (TMJ) disorder, with no resolution of the orofacial pain (4-6). This situation can lead to a delay in the diagnosis of a myocardial infarction or an angina pectoris, and subsequently the delay of the right treatment (2).

The scientific papers about orofacial pain of cardiac origin are mainly isolated clinical cases. In which the pain is located only in the orofacial region all of them, except in one case that readied to other regions. The intensity of the pain was severed and the frequency was paroxysmal in all cases. The appearance of the pain was related to physical exercise in some cases, being spontaneous the most of them. In three cases unnecessary dental treatment were performed; but the pain is relieved after the angioplasty surgery or the administration of vasodilators. The only multicentric study published until today is the one published by Kreiner et al. in 2007 (10). This study aimed to determine the prevalence of the orofacial pain in a sample of 186 patients who suffered from cardiac ischemia and to describe the location and the irradiation of the pain. But other important characteristics to identify the orofacial pain of cardiac origin are not described in that study, such as the frequency, the intensity, the precipitating factors and the situations that relieves the pain.

The purpose of this paper is presented the clinical characteristics of the orofacial pain of cardiac origin in a group of patients visited when taking a treadmill exercise test, at the cardiology service of the Can Ruti Hospital in Badalona (Barcelona, Catalonia, Spain).

## Material and Methods

The sample of that study included patients visiting when doing a treadmill exercise test, at the cardiology service of the Can Ruti Hospital in Badalona (Barcelona, Spain), diagnosed of acute myocardial infarction or angina pectoris, over a period of five months (between February and May 2009). Patients called at the treadmill exercise test for other reasons were excluded of the study. Finally, the resulting sample counted 30 patients.

A clinical questionnaire was designed in order to evaluate the patients of the sample. Patients were asked about the last episode of cardiac pain. The questions they had to answer consisted in the following as variables of that pilot study: 1: Sex; 2: Age; 3: Pain location; 4: Irradiation, 5: Intensity; 6: Frequency; 7: Oral Cause; 8: Dental treatment; 9: Resolution of the pain after dental treatment; 10: Final diagnosis; 11: Cardiac treatment; 12: Resolution of the pain after cardiac treatment.

The questionnaire was applied before and after the test, excepting the data extracted from the case history of the patient. The questions asked before had the aim of detecting any orofacial pain suffered previous the seizure, and the questions asked after the test had the aim of detecting any anginous pain in the orofacial region throughout the test.

## Results

The average age of patients was 58 years old with a range from 48 to 76 years old. Eight patients were women and 22 were men. Eleven of the 30 patients studied referred orofacial pain previously or during the cardiac seizure. Three of the eleven cases were female from 64 to 65 years old, and eight were male from 49 to 74 years old. Significant differences in male / female ratio were not identified. The location of the pain was in all cases bilateral mandibular and did not irradiate to other structures. The intensity of the pain varied from slight to severe. The frequency of the appearance of the pain was paroxysmal in eight cases and constant in three cases, and the duration was from a few hours to a maximum of 14 days. None of the cases presented signs of dental pathology as a possible cause of the pain, and none of those patients received dental treatment. On the other hand, all cases received cardiac treatment with the resolution of the symptoms. ( Table 1). Just one patient presented mandibular pain during the treadmill exercise test. This patient went in the hospital the same day as a result of presenting an unstable angor ( Table 2).

**Table 1:**
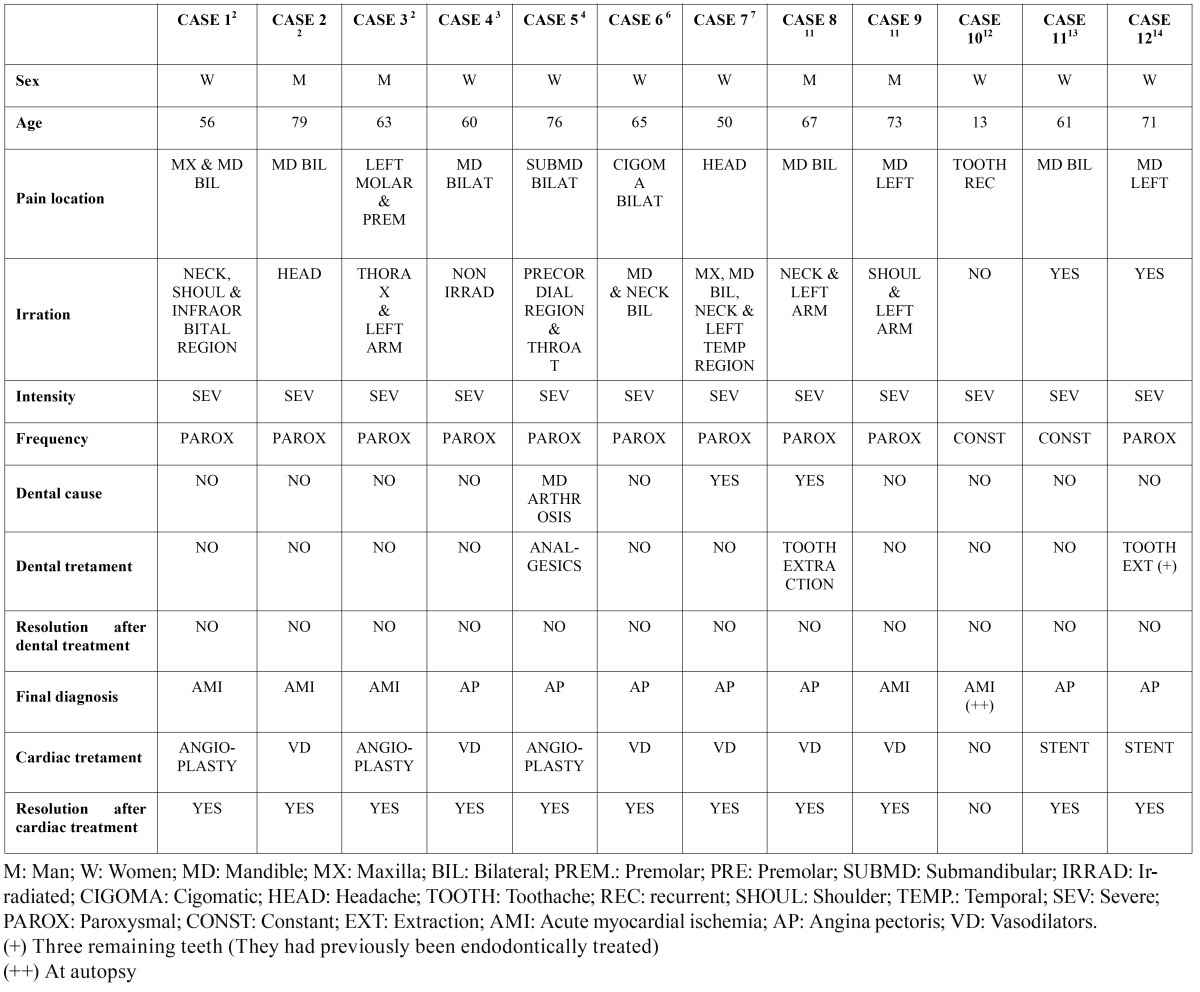
Cases reviewed in the literature about orofacial and craniofacial pain of cardiac origin.

**Table 2:**
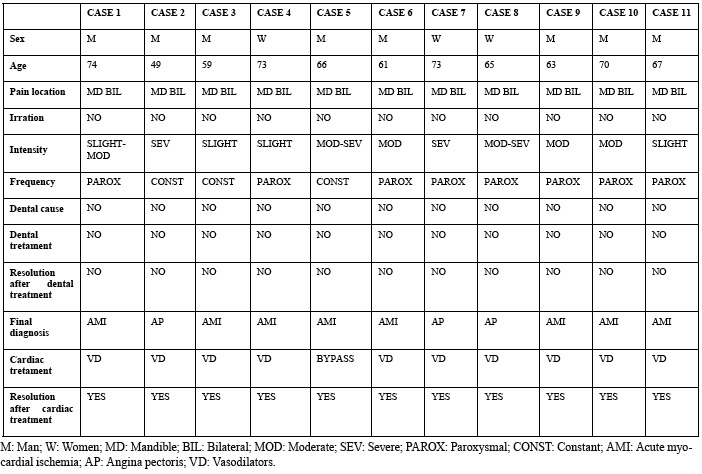
Orofacial and craniofacial pain of cardiac origin cases evaluated in this study.

## Discusion

As we mentioned above, the pain of cardiac origin that starts in the orofacial region can irradiate to the throat, neck, temporal region, head, infraorbital region, maxilla and mandible, or to the thoracic structures (thorax, shoulders and arms) (3-7,11,13). The most frequent location described in the orofacial region is the throat and the mandible (11). Other orofacial locations where the pain can be referred are: neck, maxilla, cigomatic arcs, head, TMJ, ears regions and teeth (2-4,6,7,11-14) (Table 1).

The cardiac pain referred at the craniofacial structures is usually bilateral compared to the odontogenic pain, which is always unilateral. We found that the pain is bilateral and non-irradiated in all cases.

From the twelve cases reviewed in the literature, four were males from 63 to 79 years old and seven were females from 56 to 76 years old. One case was a 13 year old female patient. The location of the pain was just in the orofacial region, referred in the maxilla, mandible, head, cigomatic arcs, submandibular region, neck, temporal region and teeth. In all cases except one, the pain irradiated to other zones such as neck, shoulders, below eye region, thorax, precordial region, throat, and to the temporal region. The intensity of the pain was severe in all cases reviewed. Ten of them had a paroxysmal frequency. Three cases received unnecessary dental treatment because of a wrong diagnosis, and consequently without the resolution of the pain. Two of them suffered unnecessary dental extractions and the other one was wrongly diagnosed temporomadibular joint dysfunction as the cause of the pain. In two of the twelve cases reviewed the appearance of the pain was related to physical exercise, in the other ten cases the appearance of the pain was spontaneous.

The evolution in time of the symptoms is just specified in 5 cases in the literature, and its duration is from 3 days to 9 months. The treatments that relieved the pain were the administration of vasodilators for 6 patients, and the angioplasty surgery for 5 patients. One patient didn´t receive any treatment, resulting in the death of that patient for cardiac causes, diagnosis that was obtained in the autopsy. After the right diagnosis of the heart pathology and the proper treatment established, a complete resolution of the symptoms was observed in all cases. ( Table 1) (2-4,6,7,11-14) .

In our serie of clinical cases, nine of 30 patients presented orofacial pain while cardiac pain, 36%. According with Kreiner et al. (10), they present 32% with pain in the craniofacial region as well as in other regions. None of the eleven cases evaluated with orofacial pain presented irradiation to other anatomical structures in our study. They present 6% with orofacial pain as the only symptom during the ischemic process. One of the eleven patients in our series presented an oral pre-cardiac episode (3%). The frequency of the pain in our study is paroxysmal in 8 cases and constant in 3 cases and the intensity varies from slight to severe. On the other hand, in the twelve cases reviewed in the literature the intensity is severe for all of them ( Table 1).

The orofacial pain of cardiac origin is considered as atypical because of its location. Other locations are rare (15). However, studies such as the aforementioned concluded that 1 out of 15 patients who suffered from a cardiac ischemia presented pain in the orofacial structures (10). Considering that the cardiac ischemia is one of the main causes of death in adult population, there is obviously an undervaluation of the clinical signs considered as atypical.

The explanation of the relationship between orofacial pain and pain of cardiac origin is based on the one hand, in certain cortex location responsible for codifying the intensity of visceral pain and in the bilateral cortex locations processing the pain (16,17). On the other hand the convergence of somatic and visceral impulses at the Central Nervous System (CNS), including the trigeminal nucleus (18,19) and the processes of central sensitization (17,19,20).

Finally, the differential diagnosis of the odontogenic pain (dental and periodontal) and the non-odontogenic pain (muscular, psychogenic, neuronal, cardiac, sinusal and neurovascular) is important in order to avoid a wrong diagnosis in the dental practice and the subsequent unnecessary dental treatment. Consequently, deeper studies evaluating an statistically significant number of patients are important to obtain a better knowledge of the orofacial pain of cardiac origin, in order to avoid unnecessary dental treatment such as tooth extraction, or TMJ dysfunction therapies, as well as accelerate the appropriate diagnosis of the coronary pathology (4,6).

## Conclusions

The conclusions of our case series we believe should be considered a provisional basis and are: -The orofacial pain of cardiac origin is located in the mandible, bilaterally, with no irradiation to other structures, being the intensity from slight to severe and the frequency paroxysmal or constant. -The treadmill exercise test is a precipitating factor of the pain. -The symptoms of the orofacial pain of cardiac origin decrease with the administration of nitroglycerine tablets or with cardiac surgery.
